# Using unique surface patterns of injection moulded plastic components as an image based Physical Unclonable Function for secure component identification

**DOI:** 10.1038/s41598-018-22876-8

**Published:** 2018-03-16

**Authors:** Benedikt Wigger, Thomas Meissner, Alexander Förste, Volker Jetter, André Zimmermann

**Affiliations:** 1 Hahn-Schickard, Institute for Micro Assembly Technology, Stuttgart, Germany; 2 Fraunhofer Institute for Physical Measurement Techniques, Freiburg, Germany; 3 University of Stuttgart, Institute for Micro Integration, Stuttgart, Germany; 4 Present Address: Balluff GmbH, Neuhausen a.d.F., Germany

## Abstract

A Physical Unclonable Function uses random and inherent properties of a physical entity and can be used to uniquely identify components e.g., for anti-counterfeiting purposes. In this work we demonstrate that the surface patterns of injection moulded plastic components themselves are inherently unique and hence can be used as a PUF for reliable and secure identification. We further demonstrate that these unique surface patterns are easily accessible since they can be photographed with a simple camera set-up. This is exemplarily demonstrated for two different plastic materials on an overall of 200 injection moulded components. A set of brief experiments further examines the PUF’s robustness towards real life conditions. This approach might be useful for secure identification and authentication of components or a label-free tracking.

## Introduction

A Physical Unclonable Function (PUF)^1,2^ is based on small variations in a component’s or product’s properties caused by small variations during its manufacturing process. Like each human being has unique individual biometric characteristics such as fingerprints, these variations can give a physical entity inherent, unclonable properties that can be used for the identification of a component.

With an ever increasing interest in security and anti-counterfeiting, literature provides numerous examples for PUF constructions for various fields of applications^3^. Prominent examples range from electronics based intrinsic PUFs^4^ e.g., for secure hardware authentication and key generation in integrated circuits and embedded systems^5,6^ to optical PUFs which e.g., exploit reflective particle tags and their random speckle patterns^7^.

Recent studies on optical PUFs focus on the manufacturing of unclonable tags, which can be attached to a product and read by optical or image-based techniques^8–13^. However, there are many cases where it is not feasible to add an additional tag to an item, be it for economic reasons, i.e. the price of large volume products or for practical reasons as it may simply not be possible to apply an additional tag to a product (e.g., for hygienic reasons in the case of medical instruments). Optical or image-based approaches that exploit the inherent patterns of a surface are ideal for such applications and have been primarily demonstrated on samples like paper^14–18^, metals^18–21^ or more exotically on melons^22^. Although some works propose to expand the approach on other materials^18^, these proposals refer to a single surface image of each material but lack any measurement and statistical basis.

Injection moulded plastic components are widely used as cost-efficient products with an estimated yearly turnover of 162 billion USD in 2020^23^. As adding an identification tag would often be more expensive than the actual manufacturing price of an injection moulded component, a purely image-based secure identification approach would be extremely valuable. However, very little research has been carried out in that direction so far. Here we present a statistical analysis of two different thermoplastic materials, *LCP Vectra E840i LDS* and *PPA Vestamid HTPlus 1031 LDS black* with respect to the suitability of their surfaces as a security feature. The major strength is that the material as well as the injection moulding process do not need to be modified making the approach easily applicable and a new approval of existing standards unnecessary. Our results show that all components can be safely identified but the identification reliability is strongly dependent on the choice of material and we further propose a simple as well as encompassing framework to test the suitability of the surface of other plastic materials.

## Results

### Samples and image capturing

**Figure 1:**
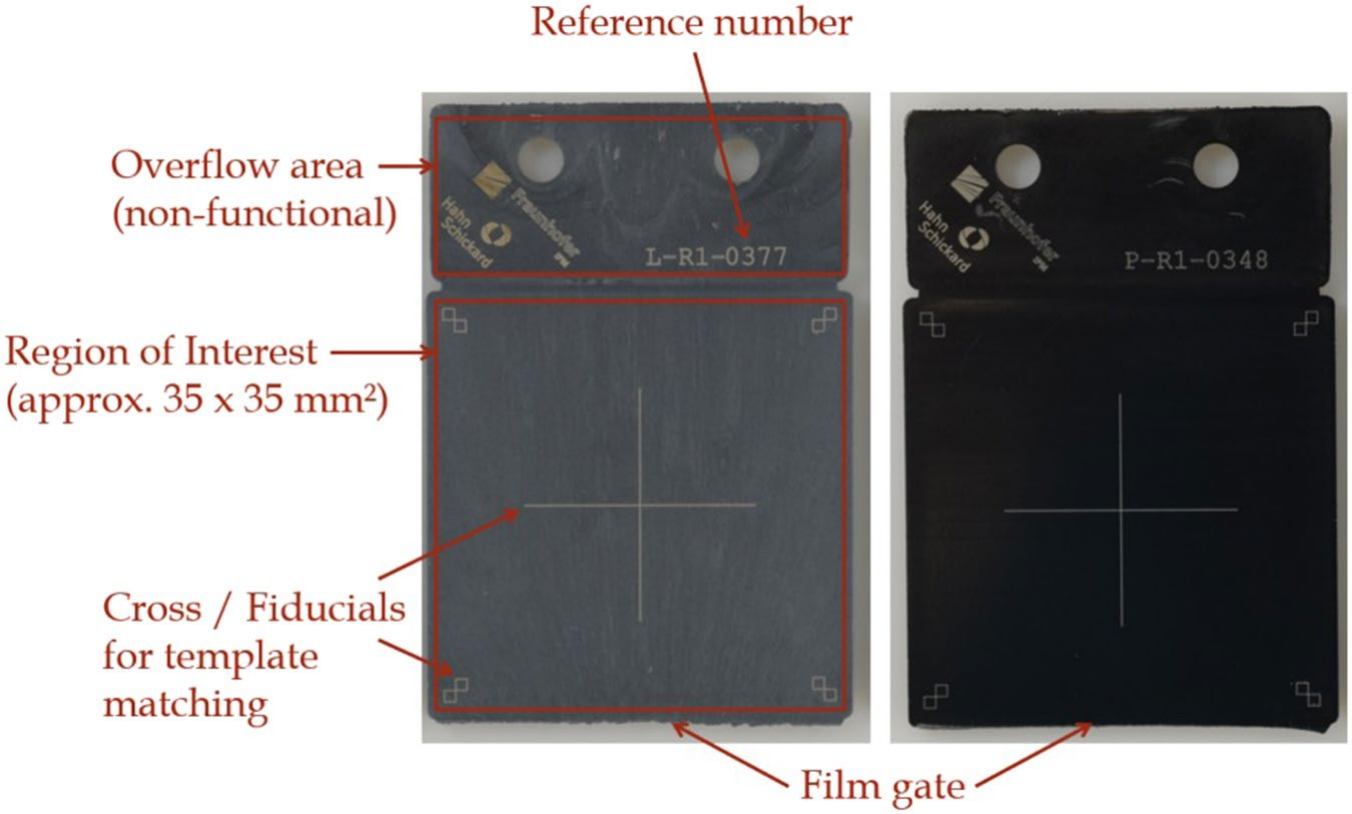
Exemplary injection moulded LCP and PPA platelet photographed by a standard reflex camera without any special lighting. On the LCP surface are flow patterns visible on first glance, whereas PPA appears homogeneously black. The overflow area increases the surface quality within the region of interest that is photographed.

From two different thermoplastic materials, *LCP Vectra E840i LDS*^24^ by Celanese and *PPA Vestamid HTPlus 1031 LDS black*^25^ by Evonik, a total of 200 (100 + 100) injection moulded samples were investigated. For each material, all samples were manufactured in a single fabrication run using an injection mould with a polished surface. Figure 1 shows one exemplary platelet of each kind. LCP, short for *liquid crystal polymer*, is a so-called high-performance thermoplastic material which is mostly applied in electronic components and frequently used at our institute. Due to the fast growing field of miniaturized electronics, the demand for LCP is expected to increase by a compound annual growth rate of 8.6%^26^. The surface appearance of *LCP Vectra E840i LDS* is gray and reveals non-homogeneous, random flow patterns that are visible to the naked eye and predestined to be used as object fingerprint. Due to its surface attributes, this LCP has been chosen to develop the tools of examination and to acquire first results. The second material under investigation is *PPA Vestamid HTPlus 1031 LDS black*, which is a *Polyphatalmide* belonging to the family of *Polyamides*. PPA materials are often used in, inter alia, automotive applications, components including light-emitting diodes, consumer products or domestic appliances^27^. At first sight, the PPA surface appears homogeneously black and does not reveal any striking contours or visible features, which is very similar to many thermoplastics in use. However, under specific lighting conditions and on closer inspection, weak surface patterns become visible. Due to their fillers, both materials are commonly used to build moulded interconnected devices.

The geometry of the test samples under study is a flat platelet design containing a main area with dimensions 37 × 37 mm^2^ as well as a so-called overflow area which is required for injection moulding to increase the surface quality in the region of interest (ROI). In the following, examinations will always regard to the surface in the ROI, whereby the considered area is about 35 × 35 mm^2^. A cross was laser etched in the center of the ROI for template matching purposes as well as a reference number in the overflow area for independent platelet tracking. It should be noted that in our experimental set up to achieve reliable and secure component identification, the ROI had to be planar in order to avoid depth of focus issues and it was kept free of coverage or shading.

For the present investigation, a commercially available industrial camera *Allied Vision Manta G-419* with 4.2 megapixels, an objective of 2.8 magnification and a monochromatic *LUMIMAX dome ring flash light* emitting in the visible red spectrum (peak wavelength at 625 nm) was used. Each platelet was photographed twice. For this purpose, it was inserted into a custom sample holder, photographed, removed, re-inserted and photographed again. Every image is edited via a template matching algorithm detecting and correcting alignment mismatches due to rotation and translation of the platelet (further information are provided in the Methods section). We define the database containing the first set of photographs of the platelets as database 1 and the database containing the second set of photographs as database 2.

The original image covers more than the ROI and has a resolution of 2048 × 2048 pixels. As a consequence of applying the template matching algorithm, the resolution of the ROI image decreased to 1800 × 1800 pixels corresponding to a ROI 35 × 35 mm^2^ in size. Using an OpenCV image processing library in Python^28^, all ROI images were converted to binary images under the use of an adaptive Gaussian threshold and further a non-local means algorithm reduces noise, both to emphasize contours and decrease the effect of inhomogeneous illumination in the case of PPA images.

**Figure 2:**
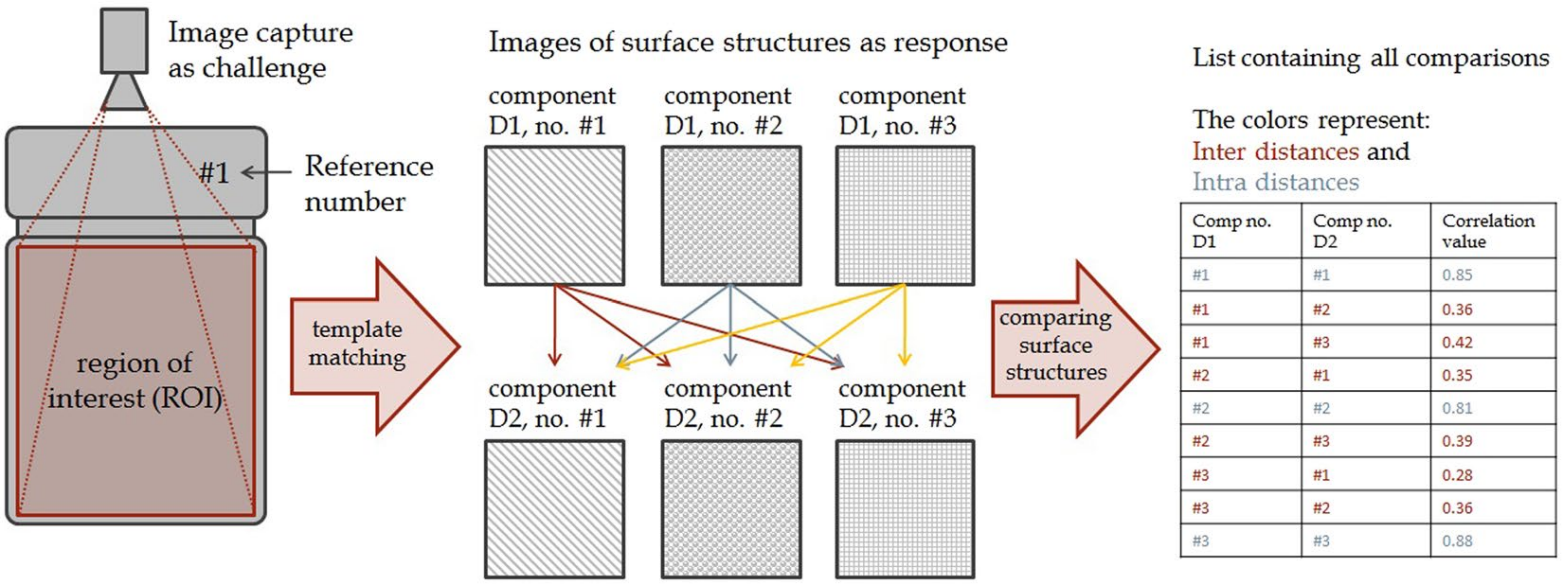
Schematic representation of the procedure in this work studying the uniqueness of injection moulded thermoplastic surface patterns. Every sample is photographed in the ROI, the images are subsequently edited via a template-matching algorithm as well as basic image processing functions. From the cross correlation of the images in database 1 with every image in database 2, one receives a list containing all correlation coefficients.

Each edited image of database 1 was then compared to the edited images of database 2 via a cross correlation algorithm provided in OpenCV (further information is provided in the Methods section). A correlation between two images is hence caused by the correlation of two components’ visible surface patterns. The algorithm yields correlation coefficients with values between 0 (no correlation) and 1 (identical surface). One therefore receives a list of correlation coefficients for each platelet. Figure 2 illustrates the process steps that have been applied to study the uniqueness of injection moulded plastic component surface patterns.

In the following discussion, *intra distance* indicates the correlation between two different images of the same platelet. For the correlation coefficient of two images taken from non-identical components the term *inter distance*^3^ will be used.

**Figure 3:**
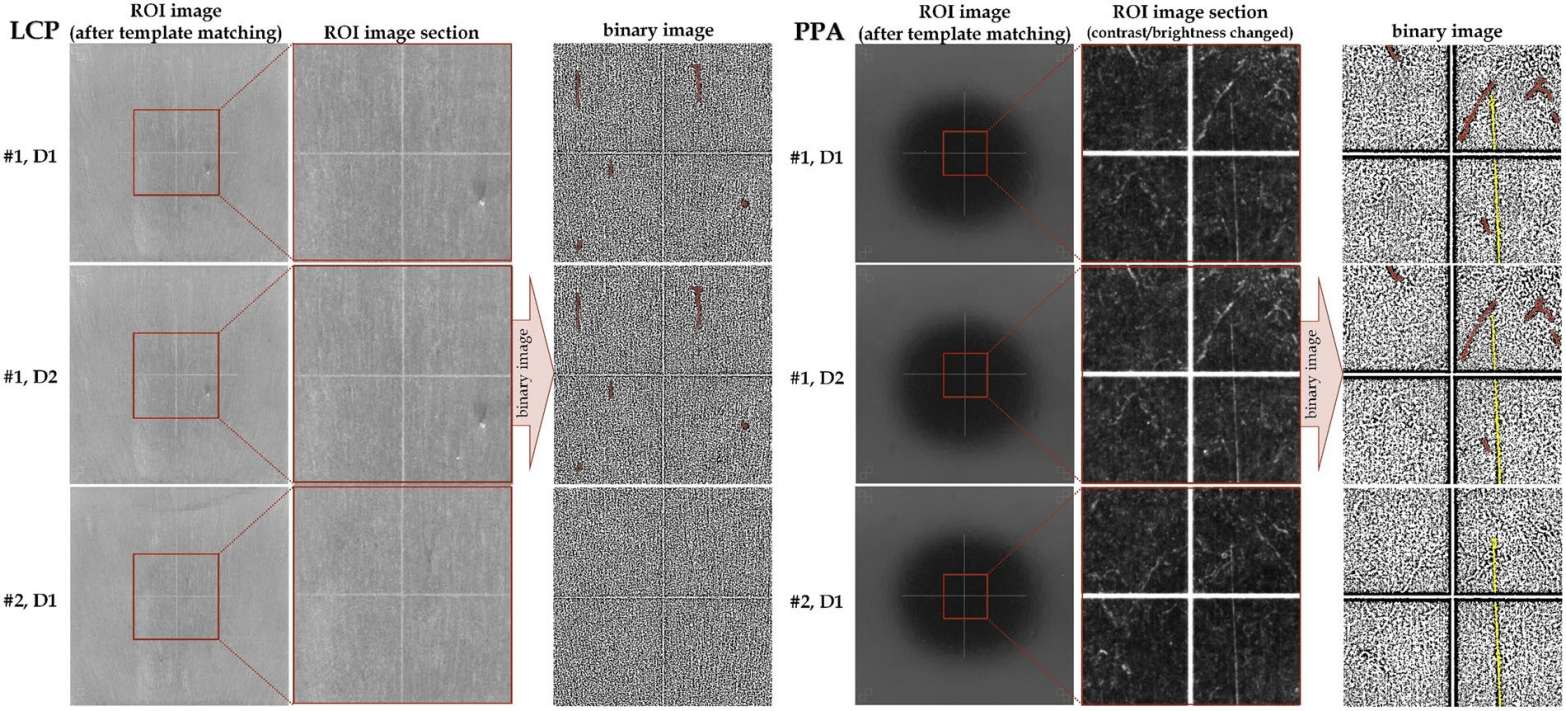
Exemplary images of both LCP and PPA components: The first two rows of images refer to only a single component, but the images are from separate records. The third row of images shows the surface patterns of a different sample. The columns show from left to right: The ROI image as it is extracted from the template matching algorithm; a smaller section of the ROI image to emphasize the individual surface patterns to the naked eye, whereby contrast and brightness in the PPA images have been modified; the identical image section, but as binary image with highlighted contours (red, yellow). The non-homogeneous illumination of a PPA platelet’s surface is due to the dome illumination set-up.

Photographs illustrating the surface patterns of LCP and PPA samples are shown in Fig. 3. From left to right, the first image column shows photographs, that are a result of the template matching algorithm and illustrate the complete ROI. The second column is intended to emphasize surface patterns in the original image to the naked eye by zooming on a smaller section of the ROI and in the case of PPA modifying contrast and brightness. Converting that section to a binary image, based on an adaptive Gaussian threshold, yields the images in the third column. Since the photographs in the top and the middle line are two separately recorded photographs of one sample, they reveal equal contours that can be used for component identification (a few have been highlighted red). Except of a scratch (yellow highlighting) that is identified on the surface of both PPA samples and, hence, is probably caused by injection-mould replication, the exemplary photographs demonstrate individual surface patterns of each sample.

Further analysis of LCP samples are based on the surface patterns revealed in the complete ROI image (left column in Fig. 3). Due to non-homogeneous surface illumination of PPA samples, however, the examination considers a smaller section of the ROI image that has 1441 × 1441 pixels corresponding 28 × 28 mm^2^ in size. Since the illumination causes contours in the outer region of the image being captured weakly or not at all, analysing only a section of the ROI enlarges the identification certainty for PPA.

### Analysis

**Figure 4:**
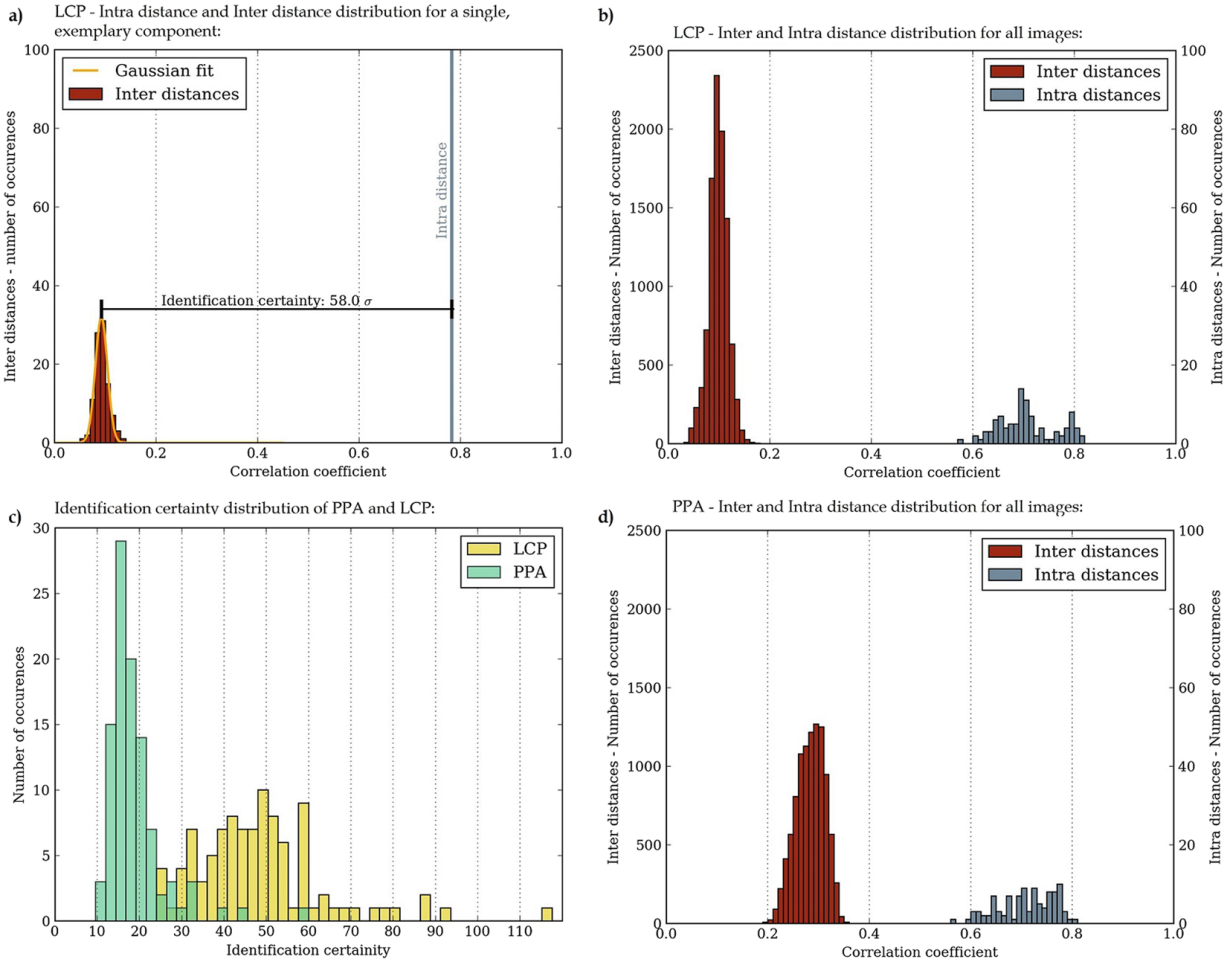
The diagrams demonstrate unique surface patterns for both LCP and PPA: (**a**) Intra distance and inter distance distribution as well as fitted inter distance probability function referring to a single LCP component. (**b**) Intra and inter distance distribution with respect to the entire LCP databases. (**c**) Histogram of the identification certainties of LCP and PPA. (**d**) Same as (**b**), but with regard to PPA.

According to the examination scheme introduced in Fig. 2, every platelet image in database 1 was compared to 100 platelet images from database 2 yielding one intra distance and 99 inter distances. Figure 4(a) illustrates the observed distribution of correlation coefficients for a single platelet of LCP. The data for the inter distance are further fitted using a Gaussian distribution in accordance with^3^. We observe a clear gap of about 0.7 in between the inter distances (non-identical platelets) and the intra distance (identical platelet). This individual platelet could therefore be safely identified. We note that with respect to the diagram, the gap size and inter distances standard deviation are the relevant parameters that have an impact on the identification certainty of a component. In Equation 1 we define the difference between the inter distances mean *μ*_inter_ and the intra distance *α* in units of the inter distances standard deviation σ_inter_ as a number representing the identification certainty *ζ*_*n*_ of a specific component *n*:

1 $${\zeta }_{n}=\frac{{\alpha }_{n}-{\mu }_{{\rm{inter}},n}}{{{\rm{\sigma }}}_{{\rm{inter}},n}}$$

This analysis uses the fitting output to determine *μ*_inter_ and σ. The exemplary sample in Fig. 4(a) is identified with a certainty of 58.0σ, whereas averaging over all LCP samples gives a certainty of 48.4σ ± 15.2σ. To put this into perspective, based on the assumption of a Gaussian distribution, the probability of an inter distance at *μ*_inter_ + 10 · σ_inter_ is below 10^−22^. Figure 4(b) shows the resulting histogram for all LCP platelets and it is evident that the inter and intra distributions are clearly separated. Hence, the results demonstrate unique surface patterns of *LCP Vectra E840i LDS* components and, based on that, a successful and very robust identification of every platelet.

The process of examination described with LCP was then repeated for PPA. Figure 4(d) shows the inter and intra distance distribution by the comparison of all PPA platelets. Due to the weaker observable surface contours in PPA, the inter and intra distances are closer to each other and the inter distances standard deviation increases. However, we still observe a clear gap between inter and intra distances and hence every platelet of PPA could be safely identified. Since the mean identification certainty is 18.9σ ± 6.9σ, even weaker contours on PPA surfaces are sufficient to achieve a very robust component identification. By optimizing the illumination set-up the identification of PPA samples is expected to become even more robust.

The histogram in Fig. 4(c) summarize the identification certainties of all samples examined in Fig. 4(b,d). The plot demonstrates that the minimum identification certainty of PPA and LCP is 9.7σ and 25.1σ respectively. The distribution of PPA samples is clearly limited to a smaller interval than the one of LCP. Although the identification certainty values are accumulated for each material in a specific range, one can observe significant higher identification certainties for a few single samples, primarily for LCP. Each of them is caused by a relatively small inter distance standard deviation.

## Discussion

The above results demonstrate that in total 200 (100 + 100) exemplary samples from two injection moulded materials, namely *LCP Vectra E840i LDS* and *PPA Vestamid HTPlus 1031 LDS black*, possess inherent, unique surface patterns. Due to the minimum identification certainty of all examined samples of 9.7σ, the component identification is very reliable. Since injection moulding is a chaotic process, it is impossible to control the injection moulding parameters to replicate a template surface. The surface patterns are therefore neither reproducible by injection moulding nor predictable, what makes the approach very robust against counterfeiting attacks as well as very appropriate to achieve secure component identification. Attempting to create a cloned surface, e.g. by transfer printing, would require other materials than the raw plastic which makes the counterfeiting provable. Achieving secure identification of common materials in a common manufacturing process without additionally manipulating the component makes the approach so valuable.

Considering potential future applications of the PUF, e.g. as alternative to RFID or labelling for component traceability or using inherent, identifiable surface patterns as security feature, the PUF’s robustness against real life conditions is essential. An additional set of brief experiments therefore addressed issues how modifications of the injection moulding process, variations of the image capturing set-up as well as impacts on the surface of a sample affect the identification certainty. Every examination is based on the procedure in Fig. 2 determining the identification certainty of two datasets of images. In a few cases one parameter had to be modified during the image post processing.

Initially, focusing on the injection moulding process, further platelets of *LCP Vectra E840i LDS* and *PPA Vestamid HTPlus 1031 LDS black* under various conditions in several runs. Changing the surface roughness of the injection mould from a mirror-polished surface to a grinded surface that obtains obvious scratches to the naked eye does not affect the identification certainty of LCP platelets, whereas the identification certainty of PPA platelets decreases from 18.9σ ± 6.9σ in Fig. 4 to 14.6σ ± 6.5σ. This can be understood as PPA to some extent replicates structures from the injection mould’s surface (see Fig. 3) and images of different samples show some similarities. Hence, the mean inter distance and the mean intra distance rise from 0.28 to 0.55 and from 0.71 to 0.82, respectively. Even though the roughened injection mould’s surface impairs the identification of PPA, every platelet was correctly identified having a minimum identification certainty of 4.1σ. The results are based on 65 platelets of LCP and 54 platelets of PPA.

The identification certainty’s dependency on injection moulding parameters was investigated with regard to the LCP material only. In^29^ it was demonstrated that the melt temperature, the injection speed and the mould temperature significantly affect the viscosity of molten *LCP Vectra E840i LDS black*. These parameters were varied in an appropriate range. Resulting 31 platelets and 27 platelets manufactured with respect to high and low viscosity yield 41.2σ ± 15.3σ and 41.3σ ± 17.5σ as identification certainty which is practically identical.

Investigating the impact of shrinkage as a consequence of injection moulding, an additional set of 47 LCP platelets was manufactured. Each platelet was photographed twice within the first two minutes after injection moulding as well as 48 hours thereafter, when the shrinking process is considered to be ended. One identification certainty analysis is based on the first two images of a sample yielding 42.2σ ± 17.9σ, another analysis compares the first image of a sample and its record after 48 hours yielding 39.2σ ± 15.0σ. The results therefore indicate that shrinkage referring to the LCP material has negligible impact.

Corresponding to the results, the surface patterns of the LCP platelets are neither significantly affected by the injection mould’s surface roughness nor by shrinkage or the variation of the injection moulding parameters indicating that the surface patterns are an inherent property of the material. Although fillers are added to both materials, the LCP surface patterns are clearly more distinct than the PPA surface patterns. Hence, it is assumed that distinct surface patterns are encouraged by specific fillers or by the molecular structure, e.g. the liquid crystal properties of molten LCP.

As goods are tracked at several locations within a company or even beyond, secure component identification must be guaranteed even under variable image capturing conditions. The exposure time, the aperture and the ambient light therefore were tested as variable and relevant parameters. Since the former two affect an image identically by increasing/decreasing the brightness, the analysis focuses on the exposure time only, which was reduced from default 220 *μ*s to 130 *μ*s. The investigations are based on another set of 30 platelets each, whereby the mean intra distance of the PPA decreases from 0.78 ± 0.01 to 0.66 ± 0.11 going hand in hand with a slight decrease of the identification certainty. In case of the LCP material the mean inter and intra distance remain roughly unchanged. No impact of changing ambient light was measured.

**Figure 5:**
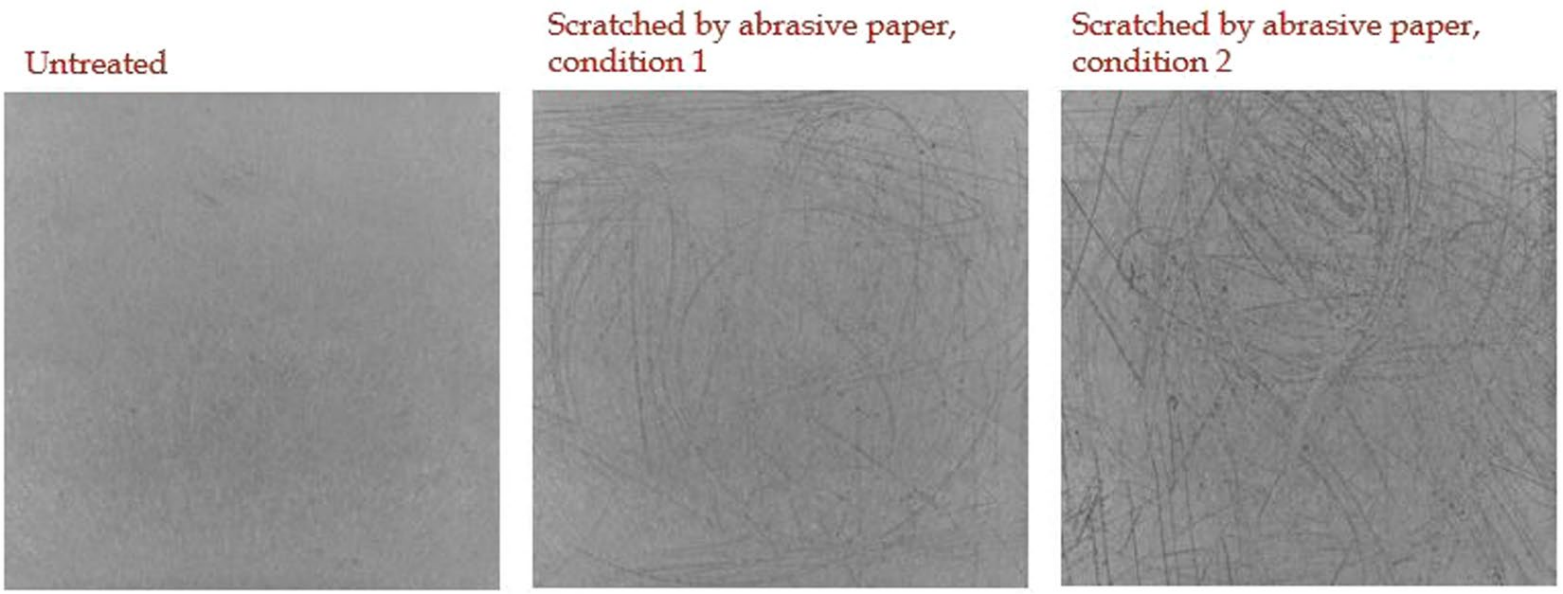
Exemplary sample of *LCP Vectra E840i LDS black*. The left image shows the untreated surface, the other two images show the platelet’s surface scratched by abrasive paper. The intra distance from left to right is: 0.65, 0.35 and 0.23. The mean inter distance is 0.15 ± 0.01.

Lastly, environmental effects, e.g. due to mechanical and chemical factors or ageing, may affect the surface patterns and therefore the identification certainty. The impact of accelerated ageing was investigated by exposing 30 platelets of LCP to 1000 thermal shock cycles between −40 °C and +125 °C. The dwell time was 10 minutes. The identification certainty of the LCP platelets alters by 2σ only indicating that the surface patterns and therefore identification are very robust. Even scratching the surface by abrasive paper does not hinder the reliable identification of a sample, although it naturally reduces the identification certainty. This was tested for one exemplary LCP sample which is illustrated in Fig. 5. From left to right the intra distance is 0.65, 0.35 and 0.23 while the untreated platelet’s mean inter distance is 0.15 ± 0.01.

The examinations based on the LCP and the PPA material demonstrate that the PUF’s performance and robustness strongly depends on the plastic material that is used. As a result, four other materials were briefly examined based on three samples each, however, without optimizing hardware or software parameters. The results are summarized in Table 1 indicating that a successful identification of larger batches of these materials is possible. In contrast, the material *PC Makrolon 2405 white* possessed a homogeneous white surface so that no identifiable patterns were available.

**Table 1:** Results of testing the PUF’s concept on four other materials based on three samples each.

Material	Mean intra distance	Mean inter distance
*PA6/6T Ultramid T LDS 4381*	0.79	0.45
*PBT Pocan DP7102 LDS*	0.34	0.14
*Tecacomp PEEK LDS*	0.75	0.49
*PA4T-GF30 - ForTii® JTX2*	0.70	0.44

Although promising results were achieved for six common plastic materials, a general statement about the PUF’s applicability to plastics is not possible as the properties of different materials vary strongly. However, the results indicate that the approach demonstrated here is useful to securely identify components of several plastic materials with anti-counterfeiting issues as well as for label-free traceability of parts and goods as an alternative to RFID or labelling. The main strength is that no modification of an existing plastic material or an adjustment of the injection moulding process is required which makes a new approval of existing standards unnecessary. Non-expensive hardware that is easy to integrate in production and expected to exhibit low operating costs would be further benefits. Furthermore, injection moulded components could be tracked from the beginning of the value chain by simply photographing a part’s surface after demoulding. Nowadays injection moulded components are usually produced as bulk material and labelled in an additional step in production. Lastly, the present technology allows the tracking of components where a label or RFID tag can not be added due to, for instance, economic or hygienic reasons. Since injection moulded components are manufactured in huge amounts, we expect their identification based on inherent surface patterns will have great potential.

## Methods

### Injection moulding

**Figure 6:**
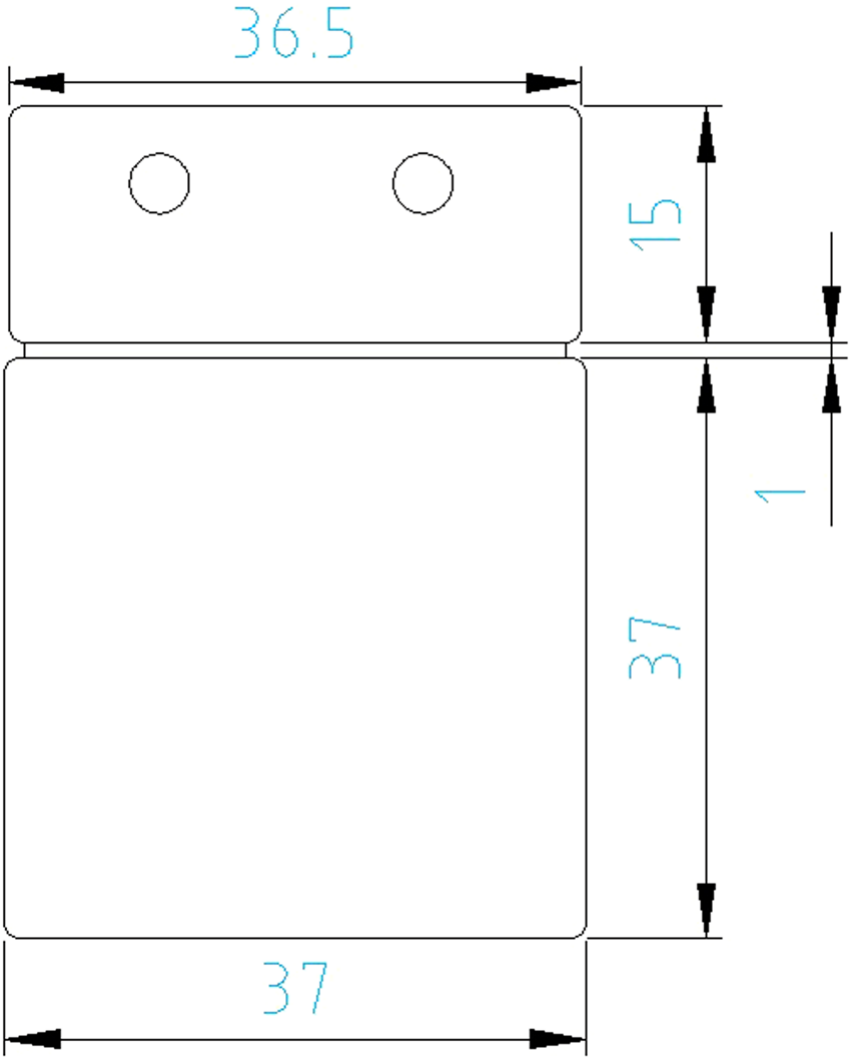
Geometry of the test platelets. The thickness is 1.5 mm.

A one-cavity injection mould was used for injection moulding test samples (Fig. 6) on an *Arburg 270A*. Injection moulding parameters were based on the materials data sheets^24,25^. The injection mould’s surface was polished.

### Laser marking

All parts were labelled with a *LPKF MicroLien3D 160i* laser system, the adjusted power was *P*_PPA_ = 2.5 W and *P*_LCP_ = 3.0 W. The cross lines have a width of 100 *μ*m and 15 mm length, both with respect to the vertical and horizontal line. Double squares in every corner of the platelet were used to verify, that the cross is centred in the ROI. They were laser-etched using the same laser settings.

### Camera and illumination set-up

The entire platelet’s area of 37 mm × 37 mm was photographed using an *Allied Vision Manta G-419* camera combined with a *Rodagon* objective having a focus length of *f* = 50 mm and a magnification ratio of 1:2.8. A *LUMIMAX Dome Flash Light* provides monochromatic red light (625 nm) at an illumination time of 220 *μ*s.

### Template matching algorithm

**Figure 7:**
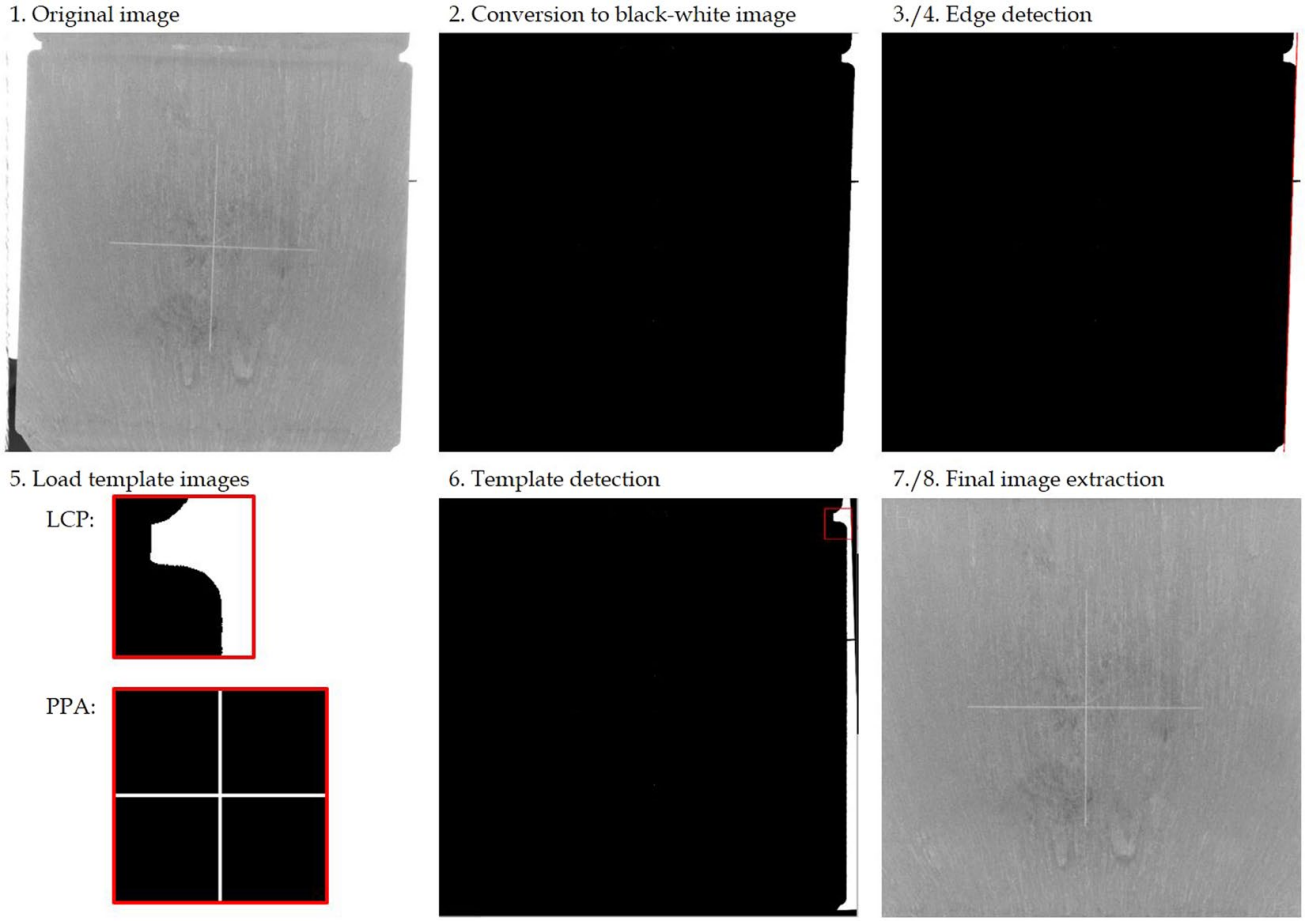
Template matching algorithm: (**1**) Load the original image. (**2**) Convert it to a black-white (binary) image. (**3,4**) Detect the platelet’s edge with Canny Edge algorithm and Hough Lines detection. This gives the rotation mismatch of the platelet. (**5**) Load the template image dependent on the material. (**6**) Detect the position of the template image. This yields information about the x-/y-shift of the platelet. (**7,8**) Extract ROI image and store it.

The template matching algorithm (Fig. 7) is applied to correct alignment mismatches of the platelet, both with respect to rotation and translation. It is implemented using *Python* and, with it, pre-implemented functions from the OpenCV-library^28^. The template matching algorithm contains the following steps:

1. Load the original image as grey-scale image.
2. Convert the original image to a binary image (black/white). For that, in the function cv2.threshold we use a global threshold dependent on the material. Besides, all pixels in the left third are defined to be black.
3. In order to detect edges like the cross on the surface or the platelet’s edge itself, we apply the Canny Edge detection^30^ to the binary image using the function cv2.canny.
4. Hough transformation^31^ yields the relevant linear equations of edges and, with it, the twisting angle of the component. The result comes from the function cv2.HoughLines. The binary image is rotated according to the twisting angle.
5. Load a template image containing black-white contours that can be identified in every binary image of PPA or LCP platelets. In the case of PPA the template image contains the cross label. Due to weak contrast of the cross label on a LCP platelet, in this case the template image contains the right edge at the passage from the main area to the overflow area.
6. Detect the template image in the rotated binary image from 4. and save the x- and y-position. This is based on the template matching function cv2.matchTemplate using the method cv2ccoeff_normed.
7. The steps above yield both the rotation angle and the x-/y-shift of the platelet. Hence, the image can easily be rotated and the ROI is subsequently extracted according to the x-/y-shift.
8. Finally, the resulting image is stored separately. Since the proceeding extracts a section from the original image, the resolution is decreased from 2048 × 2048 pixels to 1800 × 1800 pixels.

### Image post processing - adaptive thresholding and denoising

The image resulting from the template matching algorithm is processed in order to emphasize patterns and decrease the impact of non-homogeneous illumination in PPA images. Function parameters have been determined experimentally for each material in order to gain best results:

1. Load the original image as grey-scale image.
2. Convert the image to a binary image. However, this time we use the function cv2.adaptiveThreshold with the method cv2.adaptive_thresh_gaussian in order to reduce the impact of unbalanced illumination.
3. Reduce noise in the binary image using the function cv2.fastNlMeansDenoising.
4. Store the resulting image.

Applying the template matching algorithm and the image post processing to all together 200 component images yields a runtime of 431 seconds. The hardware used was a 64-Bit-Windows 7 desktop PC consisting of an *Intel Core i5-4590 CPU* with 3.30 GHz, 8 GB RAM and a standard magnetic hard disk. The runtime scales linearly with the number of component images.

### Correlation between images

In this study we compare an image from database 1 (*I1*) to an image from database 2 (*I2*). Initially, we extract 90% from the center of *I2* as template image and pass it to the template matching function cv2.matchTemplate with method cv2.ccoeff_normed, that we already used in the template matching algorithm. The result is an array, from that the maximum value is considered as correlation coefficient.

Comparing 100 images from database 1 to 100 images from database 2 takes 1116 seconds to finish all *n* = 10,000 comparisons. The runtime increases linearly with the number of image pairs to be compared *n*, whereby *n* depends quadratically on the number of components *N*: *n* = *N*^2^.

Significant improvements with respect to the algorithms’ runtime can certainly be achieved. As an example, all implemented program code is executed on only a single processor core.

### Availability of data

The datasets generated and analysed during the current study are available from the corresponding author on reasonable request.
